# Risk maps for range expansion of the Lyme disease vector, *Ixodes scapularis*, in Canada now and with climate change

**DOI:** 10.1186/1476-072X-7-24

**Published:** 2008-05-22

**Authors:** Nicholas H Ogden, Laurie St-Onge, Ian K Barker, Stéphanie Brazeau, Michel Bigras-Poulin, Dominique F Charron, Charles M Francis, Audrey Heagy, L Robbin Lindsay, Abdel Maarouf, Pascal Michel, François Milord, Christopher J O'Callaghan, Louise Trudel, R Alex Thompson

**Affiliations:** 1 Centre for Food-borne, Environmental and Zoonotic Infectious Diseases, Public Health Agency of Canada, Saint-Hyacinthe, Québec, Canada; 2 Groupe de recherche en épidémiologie des zoonoses et santé publique, Faculté de médecine vétérinaire, Université de Montréal, Saint-Hyacinthe, Québec, Canada; 3 Laboratory for Foodborne Zoonoses, Public Health Agency of Canada, Saint-Hyacinthe, Québec, Canada; 4 Canadian Cooperative Wildlife Health Centre, Department of Pathobiology, University of Guelph, Guelph, Ontario, Canada; 5 International Development Research Centre, Ottawa, Ontario, Canada; 6 Migratory Bird Populations Division, Canadian Wildlife Service, Environment Canada, Ottawa, Ontario, Canada; 7 Bird Studies Canada, Port Rowan, Ontario, Canada; 8 Special Pathogens Division, Public Health Agency of Canada, National Microbiology Laboratory, Winnipeg, Manitoba, Canada; 9 Adaptations and Impacts Research Group, Meteorological Service of Canada, Environment Canada, Canada (Now retired); 10 Institut national de santé publique du Québec, Longueuil, Québec, Canada; 11 Department of Community Health and Epidemiology, Queen's University, Kingston, Ontario, Canada; 12 Laboratoire de santé publique du Québec, Institut national de santé publique du Québec, Sainte-Anne-de-Bellevue, Québec, Canada; 13 National Surveillance Unit, Centers for Epidemiology and Animal Health USDA APHIS/Veterinary Services, Fort Collins, Colorado, USA

## Abstract

**Background:**

Lyme disease is the commonest vector-borne zoonosis in the temperate world, and an emerging infectious disease in Canada due to expansion of the geographic range of the tick vector *Ixodes scapularis*. Studies suggest that climate change will accelerate Lyme disease emergence by enhancing climatic suitability for *I. scapularis*. Risk maps will help to meet the public health challenge of Lyme disease by allowing targeting of surveillance and intervention activities.

**Results:**

A risk map for possible Lyme endemicity was created using a simple risk algorithm for occurrence of *I. scapularis *populations. The algorithm was calculated for each census sub-division in central and eastern Canada from interpolated output of a temperature-driven simulation model of *I. scapularis *populations and an index of tick immigration. The latter was calculated from estimates of tick dispersion distances by migratory birds and recent knowledge of the current geographic range of endemic *I. scapularis *populations. The index of tick immigration closely predicted passive surveillance data on *I. scapularis *occurrence, and the risk algorithm was a significant predictor of the occurrence of *I. scapularis *populations in a prospective field study. Risk maps for *I. scapularis *occurrence in Canada under future projected climate (in the 2020s, 2050s and 2080s) were produced using temperature output from the Canadian Coupled Global Climate Model 2 with greenhouse gas emission scenario enforcing '*A2*' of the Intergovernmental Panel on Climate Change.

**Conclusion:**

We have prepared risk maps for the occurrence of *I. scapularis *in eastern and central Canada under current and future projected climate. Validation of the risk maps provides some confidence that they provide a useful first step in predicting the occurrence of *I. scapularis *populations, and directing public health objectives in minimizing risk from Lyme disease. Further field studies are needed, however, to continue validation and refinement of the risk maps.

## Background

Lyme disease is an emerging infectious disease in Canada, largely due to the expansion of the geographic range of the tick vector *Ixodes scapularis *in eastern and central Canada [1]. Up to 1997, the only population of *I. scapularis *known in Canada was that at Long Point, Ontario [2]. However, the number of known *I. scapularis *populations in Canada has risen from 1 to 13 [1] in the last decade, a period that may have witnessed the first evidence of global warming [3,4]. Global warming is anticipated to accelerate expansion of the geographic range of *I. scapularis *into Canada, provided that suitable habitat and hosts occur [5], and may influence the emergence of tick-borne zoonoses [6].

If we are to limit the impact of emerging Lyme disease on human health in Canada, the appropriate public health messages and clinical information must be issued to the public and medical practitioners and targeted to the right risk locations [1]. Early diagnosis and treatment of Lyme disease is usually simple and successful, but diagnosis and treatment of later stages of systemically disseminated Lyme disease is much more difficult and costly to the patient and health services [7]. Furthermore, targeted surveillance for *I. scapularis *ticks and *Borrelia burgdorferi *(the agent of Lyme borreliosis) is needed to identify endemic locations, as this knowledge is important in assisting clinical diagnoses [8]. For vector-borne diseases such as Lyme disease, the human populations at risk are at least in part defined by the geographic occurrence of the arthropod vectors, whose existence is tightly linked to climatic variables on a continental scale [9,10], and to suitable habitat on a more local geographic scale [11,12]. Predicting the occurrence of vectors by risk maps is, therefore, a potentially very useful tool to guide public health policy and target surveillance and intervention activities for vector-borne diseases [13,14].

In many cases, potential geographic distributions of vectors have been predicted using statistical associations between climatic or landscape variables (or their remote-sensed proxies), which are likely associated with vector survival and/or reproduction, and observed occurrence of vectors or the diseases they transmit [11-17]. However, with a greater depth of understanding of how climate and landscape variables directly affect biological process of vector survival and abundance, these relationships alone can be used to develop risk maps, a process which can be particularly useful for predicting expansion of the geographic ranges of vectors and vector-borne diseases [18].

For *I. scapularis*, we have considerable empirical information on how climate, particularly temperature, can affect the survival of the ticks, and this information has already been synthesised in a simulation model to identify relationships between temperature and *I. scapularis *population survival [10]. We also have assessed empirically how habitat in southeastern Canada may affect *I. scapularis *survival [19]. Furthermore, we have information on the routes of dispersion of *I. scapularis *from locations where the tick is endemic at present. Recently, we identified northward migrating land birds as potentially important in expanding the range of *I. scapularis *for three reasons: i) a high number (circa 2%) of the estimated 3 billion birds migrating northward into and through eastern and central Canada carry an *I. scapularis *tick [20], ii) these migratory birds are capable of surmounting geographic features (lakes, sea, mountains and areas of intensive agriculture) that are obstacles to dispersal by terrestrial hosts, and iii) the long distances that migratory birds can fly during the 2–4 days that a larval or nymphal *I. scapularis *feeds on its host [21]. Furthermore, some of the ticks carried by these birds are infected with tick-borne pathogens including several genotypes of *B. burgdorferi *and *Anaplasma phagocytophilum *(the agent of Human Granulocytic Anaplasmosis) [22]. These infected ticks pose an immediate, but low-level threat to human health in Canada after dropping off the birds and moulting into the next instar [1,23]. They could infect wildlife with these pathogens thus introducing endemic cycles of infection into newly established reproducing populations of the tick vector, which would give rise to a greater public health risk [1]. This may, however, be a process that takes some years [20]. Most *I. scapularis *ticks carried by migratory birds are nymphs, which moult into adults that feed mostly on reservoir-incompetent deer, and rarely on competent reservoir host species [20].

In this study, therefore, we aimed to develop risk maps for the occurrence of the Lyme disease vector *I. scapularis *in Canada under current climate using information on how climate and habitat may affect tick survival, and how current geographic distributions may affect tick dispersion on migratory birds. We then aimed to investigate how the geographic distribution of *I. scapularis *may change under possible future climate conditions.

## Results

Throughout the study, the geographic unit was the census sub-division (CSD) whose geographic area, geographic coordinates and population information were obtained as a coverage and spreadsheet from Statistics Canada [24]. This spatial unit is increasingly used in Canadian public health because i) it is a well defined spatial unit available to users, including provincial and municipal public health organisations, ii) its spatial boundaries are relatively stable over time, and iii) accompanying data on human population acts as a denominator for surveillance activities [23].

### Selection of risk algorithms and cut-off levels

**Figure 1:**
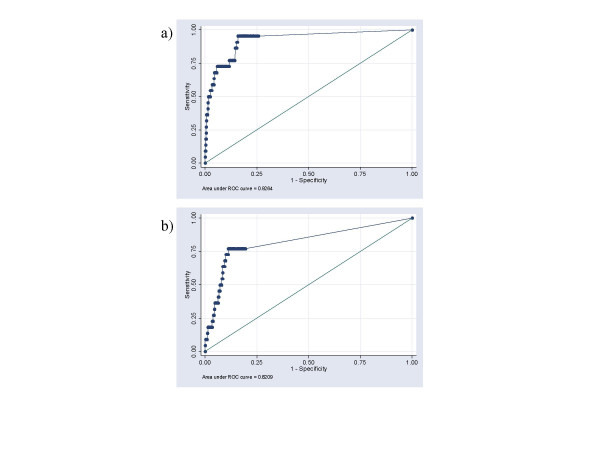
**ROC analysis of risk algorithms**. ROC graph of the relationship between sensitivity and 1-specificity for detection of known or suspected *I. scapularis *populations in Canada using Algorithm 6 (graph a: risk index = number of ticks at model equilibrium × number of tick populations within 425 km) and Algorithm 12 (graph b: risk index = number of ticks at model equilibrium × number of tick populations within 425 km × percentage forest cover).

A number of algorithms to estimate risk of establishment of *I. scapularis *were calculated from a combination of values for i) output of a simulation model of *I. scapularis *populations indicating the temperature suitability for the tick, ii) an index of the numbers of ticks immigrating on migratory birds, and iii) the percentage coverage of forest habitat (which is that most suitable for *I. scapularis *[19]). The algorithm that, in Receiver-Operator Characteristic (ROC) analysis, performed best at predicting the CSDs that are currently known to contain *I. scapularis *populations was a simple multiplication of the number of ticks at model equilibrium (predicted from DD > 0°C) and the index of nymphal tick immigration (algorithm 6). This algorithm had an area under the curve (AUC [25]) of 0.926 (asymptotic normal 95% confidence interval [CI] = 0.869 – 0.983: Table 1). Including the tick immigration index marginally, but not significantly (χ^2 ^= 0.5, P > 0.1), increased AUC compared to algorithm 1, which considered only the number of ticks at model equilibrium (for which the AUC was 0.921). However, because tick dispersion by migratory birds is likely limiting on the establishment of *I. scapularis*, the index of nymphal tick immigration was considered essential to risk mapping and its effect retained by choosing algorithm 6. Including an index of larval tick immigration did not improve this algorithm. Also, including the percentage forest cover did not improve AUC, in fact the AUC for algorithm 6 was significantly higher than that one of the best performing algorithms containing forest cover (algorithm 12, χ^2 ^= 4.25, P < 0.05, Fig 1). Logistic regression analysis supported ROC analysis and algorithm 6 showed the best fit according to Aikeke's Information Criterion (AIC) (Table 1).

**Table 1:** Algorithms used to predict the occurrence of Canadian census sub-divisions containing resident *I. scapularis *populations.

	Algorithm	AUC	SE	95% CI	AIC
1	No. of ticks at model equilibrium (*T*)	0.921	0.030	0.863 – 0.979	214
2	No. of ticks at model equilibrium categorised (*Tc*)	0.780	0.052	0.678 – 0.881	206
3	Percent forest area (*F*)	0.387	0.038	0.313 – 0.461	232
4	Index of larval tick immigration (range 255 km: *I*_*L*_)	0.816	0.052	0.713 – 0.919	211
5	Index of nymphal tick immigration (range 425 km: *I*_*N*_)	0.896	0.025	0.848 – 0.949	216
6	T * *I*_*N*_	0.926	0.029	0.869 – 0.983	180
7	Tc * *I*_*N*_	0.807	0.055	0.699 – 0.914	183
8	T * *I*_*L*_	0.845	0.052	0.743 – 0.947	207
9	Tc * *I*_*L*_	0.723	0.056	0.614 – 0.832	207
10	T * *I*_N _* (0.05* I_*L*_)^†^	0.926	0.029	0.869 – 0.983	185^1^
11	Tc * *I*_N _* (0.05* I_*L*_)^†^	0.807	0.055	0.699 – 0.914	186^1^
12	T * *I*_N_* F	0.821	0.050	0.723 – 0.919	185^2^
13	Tc * *I*_N_* F	0.752	0.054	0.646 – 0.858	186^2^
14	T * *I*_N_* Log_10 _*F*	0.832	0.051	0.732 – 0.933	185^2^
15	Tc * *I*_N_* Log_10 _*F*	0.762	0.056	0.652 – 0.871	187^2^

^†^Larva-to-nymph survival of *I. scapularis *is approximately one twentieth of nymph-to-adult survival (Ogden et al., 2005). ^1 ^In all logistic regression models containing variables relating to forest cover, these variables were not significant. ^2 ^In neither of these models was the variable (0.05* I_*L*_) significant.

The performance of different risk algorithms in ROC analysis is shown: AUC = area under the ROC curve, SE = standard error, 95% CI = 95% confidence interval for AUC. AIC = Aikeke's Information criterion of a logistic regression model for each algorithm in which the outcome was the occurrence of a known *I. scapularis *population, and the explanatory variables were the algorithm component(s).

### Validation of index of nymphal tick immigration

When accounting for human population in each CSD, the index of nymphal tick immigration was strongly and significantly associated with the 2319 *I. scapularis *ticks submitted in passive surveillance in Canada up to 2004 [23] in bivariable analyses (coefficient = 0.016, SE = 0.003, P < 0.001), and particularly strongly in multivariable analyses (Table 2). In these multivariable analyses, the inter-province variation in the numbers of submitted ticks followed the anticipated pattern: strong surveillance efforts and correspondingly higher numbers of ticks in the Atlantic provinces, Quebec and Manitoba, while low surveillance effort in Saskatchewan and (due to competing surveillance systems) apparent low effort in Ontario were associated with low numbers of ticks (Table 2[23]). A multivariable model with province and the index of nymphal tick immigration fit better than a multivariable model with province and latitude as explanatory variables (AIC = 3976 and 4048 respectively). Including both latitude and the index of nymphal tick immigration did not improve fit further (AIC = 3976). One outlying observation was the prediction that Newfoundland was beyond the range of bird-borne *I. scapularis *from known endemic sites in Canada or the USA. In fact *I. scapularis *have been found by passive surveillance in Newfoundland [23], indicating that ticks are dispersed beyond the 425 km mean dispersion distance, or suggesting that birds fly faster over water bodies, (such as the Gulf of St Lawrence), than over land from where our flight speed estimates were obtained.

**Table 2:** Validation of the nymphal tick immigration index.

Variable	Factor	Coefficient (SE)	Wald z
Index of tick immigration		0.043 (0.004)	10.86***
Province	Newfoundland & Labrador	Reference factor	
	Saskatchewan	-2.524 (0.254)	-4.12***
	Manitoba	1.692 (0.289)	5.85***
	Ontario	-1.27 (0.299)	-4.24***
	Quebec	-0.405 (0.304)	1.33
	New Brunswick	0.482 (0.303)	1.59
	Prince Edward Island	3.131 (0.315)	9.93***
	Nova Scotia	0.583 (0.349)	1.67
Constant		-9.831 (0.254)	-38.65

*** = P < 0.001

The association of the nymphal tick immigration index with the number of *I. scapularis *submitted in each Canadian census sub-division in passive surveillance in Canada from 1990 to 2003 was investigated using a multivariable negative binomial regression model. In this model nymphal tick immigration index and Province were explanatory variables, and the natural logarithm of human population was included as an offset.

There was almost complete confounding between the index of nymphal tick immigration for each CSD in southern Quebec and the latitude of the CSD, a risk factor for ticks submitted by passive surveillance that was found significant in a previous study [23]. In a minimal multivariable model in which Ln human population was an offset, average spring normalised difference vegetation index (NDVI: a second risk factor for *I. scapularis *identified by Ogden et al [23]) and the index of nymphal tick immigration were significantly associated with the number of *I. scapularis *submitted by the public (coefficients = 1.845 and 0.063, SE = 0.48 and 0.01, Wald Z = 3.8 and 8.7 respectively, P < 0.001 for both). AIC for models containing latitude and the index of nymphal tick immigration were 1362 and 1366 respectively, and with both variables together in the model, AIC was 1365, i.e. the index of nymphal tick immigration and latitude were almost totally confounded, supporting the hypothesis in Ogden et al [23] that latitude acted as a proxy for tick dispersion on migratory birds or other animal hosts of the tick.

### Risk maps under current and projected climate

**Figure 2:**
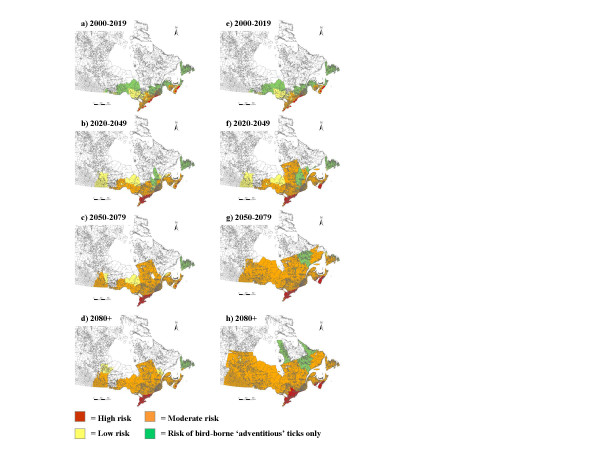
**Risk maps for the occurrence of the Lyme disease vector *Ixodes scapularis***. Expansion of *I. scapularis*-affected CSDs in Canada from the present (using 1971–2000 temperature normals) to the 2080s (using the temperature conditions predicted by the CGCM2 climate model under emissions scenario *A2*). In Figs a to d, the 'slow' scenario, the model assumes that by the end of each time period, only risk CSDs with an algorithm value in the top 10% will contain an *I. scapularis *population. In Figs e to h, the 'fast' scenario, the model assumes that by the end of each time period, all CSDs within the 'moderate' risk zone for *I. scapularis *establishment (risk CSDs) contain an *I. scapularis *population. For both scenarios, the time steps are 2000 to 2019, 2020 to 2049, 2050 to 2079 and 2080 to 2109. The 'high' risk regions for *I. scapularis *population establishment are indicated in red, the 'moderate' risk regions are in orange, the 'low' risk regions are in yellow, regions with no risk of established populations but some risk from bird-borne 'adventitious' ticks are in green, and regions with no predicted risk of either are colourless.

Algorithm 6 was the scale used to establish cut-off values for the level of risk of *I. scapularis *establishment in each CSD. Risk categories were: 'high', 'moderate' and 'low' for establishment of *I. scapularis *populations, 'risk of adventitious ticks' where CSDs were within the range for migratory bird-transported ticks but had temperature conditions unsuitable for tick population establishment, and 'no risk' where temperature conditions of the CSDs were unsuitable for population establishment, and where the CSDs were beyond the expected range of bird-transported ticks. The risk maps obtained under current climate, and future projected climate, are shown in Fig 2. Of the known tick populations, 9 were in the high risk zone, 3 in the moderate risk zone and 1 in the zone where there is no risk predicted for *I. scapularis *except from bird-borne adventitious ticks. The progression of predicted range of *I. scapularis *under future climatic conditions, from the 2020s to the 2080s and beyond, was simulated using two 'scenarios' for the rate at which new *I. scapularis *populations establish once climatic conditions and tick immigration rates become suitable for population establishment according to the risk algorithm. Under the 'slow' scenario for tick establishment (Figs 2a to 2d), tick populations only established in the CSDs considered 'high risk' during each 30 year time interval, while under the 'fast' scenario (Figs 2e to 2h), tick populations established in both 'high risk' and 'moderate risk' CSDs. Under current temperature conditions, 219 CSDs were classified as 'high risk', 1875 as 'moderate risk' and 1514 as 'low risk'. Under the 'slow' scenario for tick establishment the numbers of 'high risk' CSDs increased to 605, 758 and 809 by years 2049, 2079 and 2109 respectively, and the numbers of 'moderate risk' CSDs increased to 4307, 5669 and 6082 by these dates. Under the 'fast' scenario for tick establishment, 'high risk' CSDs increased to 699, 1065 and 1499 by years 2049, 2079 and 2109 respectively, and the numbers of 'moderate risk' CSDs increased to 4793, 6640 and 9188 by these dates. Note that the minimum value for the nymphal tick immigration index for all CSDs in Newfoundland was set at 1 because of the possibility that birds fly faster over water to reach this island than they fly over land.

### Field validation

**Figure 3:**
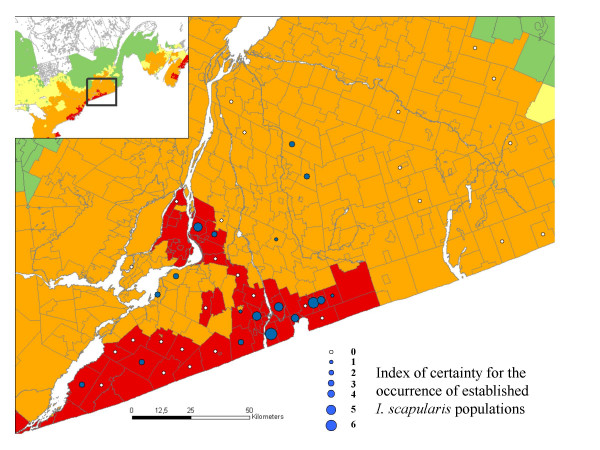
**The outcome of field validation of risk maps**. A map of southern Quebec showing the locations (unfilled or blue-filled circles) of CSDs in which field study sites were visited. The 'index of certainty' for the presence of an *I. scapularis *population was calculated from the abundance of ticks and the numbers of instars discovered during the field visit. The value 0 indicated that no *I. scapularis *ticks were found. The 'high' risk regions for *I. scapularis *population establishment are indicated in red, the 'moderate' risk regions are in orange, the 'low' risk regions are in yellow, and regions with no risk of established populations but some risk from bird-borne 'adventitious' ticks are in green

**Figure 4:**
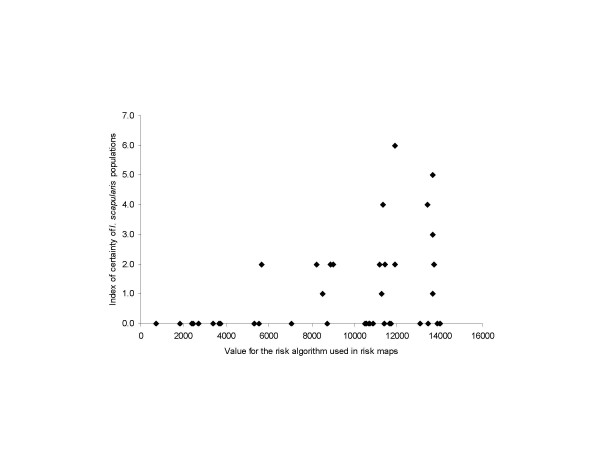
The relationship between the risk algorithm on which the risk maps were based, and the index of certainty that a site contained a reproducing *I. scapularis *population.

Forty-five woodland sites (one per CSD except in one case) were visited in southern Quebec from 5^th ^June to 5^th ^October 2007. *I. scapularis *were collected at 18 sites (40%). The numbers of *I. scapularis *found at each of these sites varied from 1 to 29, two tick instars were found at 5 sites and all three tick instars were found at two sites. Of the sites where more than one instar was found, 1 occurred in the 'moderate risk' category (5.5% of these sites), and 6 occurred in the 'high risk' category (22.2% of these sites). The proportion of sites in CSDs of the 'high risk' category where *I. scapularis *were found was higher than sites in CSDs in the 'moderate risk' category (13/27 [48.1%] and 5/18 [27.8%], Fig 3). These proportions were not significantly different but over 400 sites would have to be visited to distinguish two zones in which the true prevalence was 48% and 28%, with 80% power. However, the value of the risk algorithm chosen for mapping (algorithm 6) was significantly and positively associated with an 'index of certainty' for the presence of an *I. scapularis *population in ordered logistic regression (coefficient = 0.0003, SE = 0.0001, P < 0.01; Figs 3 and 4). The index of certainty was calculated from the abundance of ticks and the number of tick instars found at each site. The components of the risk algorithm, i.e. the number of ticks at model equilibrium and the index of nymphal tick immigration, were individually less strongly associated with the index of certainty for the presence of an *I. scapularis *population (coefficients = 0.014 and 0.079, SEs = 0.006 and 0.043, P < 0.05 and > 0.05). The model with the 'index of certainty' as the explanatory variable fit better than the models with either the number of ticks at model equilibrium or the index of nymphal tick immigration as explanatory variables (AICs = 107.5, 110.4 and 113.8 respectively). Potential confounding variables, including the date of sampling, field team and the number of *Peromyscus leucopus *and *P. maniculatus *rodents captured per site (which ranged from 5 to 49) were not significantly associated with the index of certainty for the presence of an *I. scapularis *population (χ^2 ^= 3.033 and 0.001, df = 3 and 1, P > 0.1 for both).

## Discussion

In this study, we developed an algorithm for predicting the occurrence of *I. scapularis *populations in Canada. The algorithm predicted with reasonable accuracy the occurrence of the small number of *I. scapularis *populations that were known in Canada prior to this study. A warming climate was considered to cause changes in the speed of bird migration and distances of dispersion of ticks by migrating birds, and changes in the geographic range of territory with a climate warm enough for the ticks to complete their lifecycle and establish populations. With this information, the algorithm was then used as a simple model to predict the progression of *I. scapularis *as it spreads across Canada as the climate warms, given two scenarios for the current potential occurrence of *I. scapularis *populations in Canada.

There were of course many assumptions in this process. These include i) the assumptions of the *I. scapularis *population model [10], which have more effect on the gradient of the relationship between tick abundance and DD > 0°C than on the intercept with the x-axis [10]; ii) assumptions of the climate models and the emissions scenario; iii) assumptions that the interpolation of temperature data from meteorological stations and, in particular, output from the climate model, which have a crude 400 km spatial scale, have a negligible effect; iv) assumption that the distribution of *I. scapularis*-endemic areas in the USA described by Dennis et al. [26] represents the true distribution, and that the distribution of known *I. scapularis *populations in Canada represents the true distribution; v) assumption that the speed at which birds migrate is equal across Canada irrespective of water bodies or other hazards for the birds (which is possibly relevant to the Atlantic provinces but unlikely to impact dispersion elsewhere in Canada); and vi) assumption that there are no other determinants of *I. scapularis *establishment (e.g. host densities) that are more limiting than temperature and rates of dispersion by hosts. The latter is relevant to Prince Edward Island and Newfoundland and Labrador where there are no white-tailed deer, which are thought to be key hosts for adult *I. scapularis *[22].

Our validation of the index of nymphal tick immigration suggests that the distribution of *I. scapularis*-endemic sites in Dennis et al [26] combined with the estimate of bird dispersion ranges from these and Canadian endemic areas provides a workable model of the spatial dispersion of *I. scapularis *across Canada. Furthermore, the field study in southern Quebec suggested that the risk algorithm predicts the occurrence of *I. scapularis *surprisingly well in an area where, prior to our study, reproducing *I. scapularis *populations were not thought to exist. The selected risk algorithm performed better at predicting the occurrence of potential *I. scapularis *populations than either of its two component parts individually (model-derived relationships between temperature conditions and tick abundance, and an index for predicted numbers of immigrating ticks), i.e. there appeared to be synergy between these values. This field validation, combined with validation of the index of nymphal tick immigration, gives confidence that the maps may give us insight into the possible range of *I. scapularis *at present, that temperature is a likely limiting factor on *I. scapularis *distribution, and that climate change could drive changes to the geographic footprint of this tick and possibly Lyme disease risk in the coming decades. The prevalence of *I. scapularis *positive sites in both high risk and moderate risk zones were lower than the highest estimates for prevalence of positive CSDs predicted by algorithm cut-off values. This difference could be expected as absence of *I. scapularis *from one site per CSD does not confirm complete absence of *I. scapularis *from that CSD, and because at the edge of range expansion it would be expected that not all *I. scapularis*-suitable niches are filled by *I. scapularis *populations.

Clearly, the further we project into the future the distributions of *I. scapularis *populations, the less certain we are of them because of uncertainties in emissions scenarios, climate model outputs and rates of tick dispersion. However the risk maps as presented represent both an architecture and a starting point for decision making on policies on, and targeting of, surveillance activities. Surveillance data should then be used to continue validation of the risk maps and drive refinements due to changes in the algorithm used here, or by addition of additional information as described in the following.

Our field studies suggest that the rate of *I. scapularis *establishment may be somewhere between the 'fast' and 'slow' scenarios, i.e. it is likely that not all CSDs identified as having 'moderate risk' for *I. scapularis *populations actually contain them, but some possibly do. The rate at which *I. scapularis *populations establish in climatically suitable areas, given a particular rate of tick immigration needs further field study to enhance our power to predict tick establishment. Increasing knowledge of the location of *I. scapularis *populations in Canada will also allow us to refine the relationships between climatic suitability and tick immigration rates by more formal spatial regression analyses. Furthermore, we need to investigate to what extent land mammals such as deer [27] may become more important than migratory birds in dispersing *I. scapularis *once the ticks become more widely established in south eastern Canada. Dispersion by land mammals would introduce more biologically explicit spatial autocorrelation amongst location-specific probabilities of *I. scapularis *population establishment.

Habitat, as described by percentage forest cover of CSDs was not a factor that improved prediction of current *I. scapularis *populations even though there is *a priori *evidence that habitat is an important factor for *I. scapularis *survival, and a determinant of the density of the mammalian and avian hosts of the tick [19]. It is likely that sufficient suitable habitat exists in all CSDs even if the percent coverage is small. Indeed, investigations not described here suggest that percentage forest cover could have a non-linear relationship with tick population occurrence: a high percentage of cover could mean a lot of incoming migratory birds and a lot of habitat suitable for tick survival, but small percentages of forest cover may mean that migratory birds are concentrated into finite areas with correspondingly high densities of bird-borne ticks. However, the estimates of forest cover are crude because a number of averaging steps were used, including averaging of remote-sensed AVHRR data into land cover maps and averaging of land cover maps per CSD. Therefore the forest cover estimates are likely to be crude indices of habitat suitability for *I. scapularis*, its hosts and the numbers of migratory passerines such habitats may attract.

Further studies are required to expand our current knowledge of the suitability of habitat for *I. scapularis *in Canada. Habitat may be a more useful predictor of the occurrence of *I. scapularis *at a finer spatial scale than that used here [19]. Further studies are under way in southern Quebec and elsewhere in Canada to investigate the rates at which the agent of Lyme disease, *B. burgdorferi *becomes established in newly-established *I. scapularis *populations, to determine at what rate the risk of a tick bite becomes the risk of an infected tick bite. The rate of *B. burgdorferi *establishment may depend on factors that are not explicitly considered in our algorithms at present, such as reservoir host densities, although in the initial expansion zone in southeastern Canada, the densities of reservoir hosts and adult tick hosts such as deer are not considered limiting factors [5].

Habitat may have another effect on predictions. If off-host tick mortality is higher or lower than that set in the *I. scapularis *population model, then the model can slightly over- or under-estimate (respectively) tick abundance [10,19]. The model was parameterised in woodland habitats of central/eastern Canada, and greater tick survival in woodland habitats of Manitoba is a possible explanation for the survival of the *I. scapularis *at the Manitoba site when the model predicted population die-out. In other studies, ROC analysis of the predictions of logistic regression models has been used to identify geographic variation in the predictive power of regression models [28]. Here our method identified possible regional variations in the predictive power of the risk maps, although we cannot, without field studies, rule out the possibility that the microclimate at the Manitoba site is in fact suitable for tick survival as the model predicts.

For simplicity we have only used one emissions scenario and the output from one global climate model in our study. More detailed studies await i) output from the third version of the Canadian Coupled Global Climate Model (CGCM3, which is almost ready for use by the scientific community), ii) new emissions scenarios from the fourth assessment of the Intergovernmental Panel on Climate Change (IPCC), and iii) projected climate at a much finer geographic scale courtesy of regional climate models for North America [29]. If and when *I. scapularis *becomes established in the more northern regions of risk identified by the risk maps, then a different unit of spatial scale than CSD will be needed as CSDs, which partly reflect population densities, become vary large in northern Canada.

## Conclusion

We have prepared risk maps for public health use now for identifying current and possible future areas of risk for *I. scapularis *ticks and, by inference, possible current or future Lyme disease risk. The risk maps suggest a possible widespread expansion of *I. scapularis *across southeastern, and eventually south central Canada during the coming decades, while our field study suggests that this process could already have begun. We have described a process for creating an index of risk for the occurrence of arthropod vectors of public health importance in Canada using information on the biology of vector survival and dispersion. As such, this could act as a model for assessing the spatial and temporal risks from other vector-borne zoonoses in Canada that are not currently endemic but which may become so due to climate change. Such modelling and mapping studies need, however, the support of solid field and laboratory studies. The difference in the rates and ranges of *I. scapularis *between the two scenarios for the rates of tick establishment in suitable habitat illustrates this point. More accurate prediction of where *I. scapularis *occurs now (i.e. the start point for simulations) and at what rate *I. scapularis *will establish, and are establishing in Canadian habitats, can only be elucidated using refinements of the risk maps presented here. This will require careful and more extensive field studies than those conducted here. Such studies are therefore a priority if we are to achieve our goal of limiting the impact of Lyme disease in Canada by targeted surveillance and preventive measures.

## Methods

All mapping and geospatial analyses were performed in ESRI ArcGIS 9.1 (ESRI 2004). All layers were in Canada Albers Equal Area Conic projection (datum NAD83 and ellipsoid GRS80). There were 10030 complete or partial (i.e. divided by geographic features such as rivers) CSDs east of longitude 100°W used in this study. The mean area of these CSDs, an indication of the spatial resolution of the risk maps, was 285 km^2 ^(SD = 5750 km^2^), although this value was much lower, with less variation amongst CSDs, for CSDs close to the USA border (mean 85 km^2^, SD = 277 km^2 ^for CSDs south of latitude 46°N in southern Quebec, Ontario and the Maritime Provinces).

### Risk factors for *I. scapularis *occurrence used in developing a risk algorithm

We have *a priori *information to suggest that *I. scapularis *distributions in Canada are dependent on combined effects of three key variable: ambient temperature, habitat and the numbers of immigrating ticks on migratory birds [10,19,20]. Therefore, these variables were selected as components of a risk algorithm as described in the following.

#### Temperature

In a previous modelling study, the maximum annual number of feeding adult female ticks at model equilibrium (as an index of overall tick abundance, and thus climatic suitability for *I. scapularis*) was linearly related to the cumulative annual number of degree-days > 0°C (DD > 0°C [10]). We therefore created an interpolated surface of DD > 0°C, using Environment Canada 1971–2000 temperature normals, for all Canadian meteorological stations that have at least 15 years data in this period. Data from 2368 meteorological stations were used and the mean distance between these stations for the whole of Canada was 24.6 km (SD = 36.8 km), although in southern Canada the mean distance was much smaller (15.4 km, SD = 12.6 km). Exact local deterministic interpolators (Radial Basis Function [RBF] and Inverse Distance Weighted [IDW]: [30,31]) were used to interpolate the values and differences between the methods investigated. The best statistical results (mean and root mean square of predictions errors) were obtained with RBF (thin-plate spline function). However, RBF predicted values above the maximum and below the minimum measured values. In particular, RBF produced positive values for the number of ticks > 1 in 'ripples' in areas beyond the northern edge of predicted regions of climate suitability for the ticks, i.e. where all surrounding recorded values had DD > 0°C value below the threshold for tick population survival. We considered that this was a problem that was important to eliminate in our study because we wished to identify areas that are predicted to be unsuitable for *I. scapularis *establishment as well as those that are predicted to be suitable. IDW was the method chosen because it is a basic interpolation method that predicted values inside the minimum and maximum value range and also because the data were normally distributed. We did not account for altitude in interpolations because wide variations in altitude between meteorological stations (as occur in western Canada where they must be accounted for [18]) are uncommon in central and eastern Canada except in Labrador and to a lesser extent the northern end of the Appalachian chain in Quebec. In these regions, incorporation of altitude would be necessary in finer scale risk maps for targeting surveillance activities.

The maximum DD > 0°C value within each CSD was assigned as the DD > 0°C value for that CSD and then converted to the expected number of ticks at model equilibrium using the relationship in reference [10]. The maximum value was used to avoid misclassifying CSDs as wholly unsuitable for *I. scapularis *establishment when they could actually contain *I. scapularis *populations at least at some locations. We used the relationship between DD > 0°C and the number of ticks obtained in the *I. scapularis *population model obtained when using temperature data from locations in southern Ontario [10]. Below a threshold minimum value of 3063 DD > 0°C, deterministic die out of tick populations occurred in model simulations. As a result, for values of DD > 0°C at or below this threshold, the number of ticks at model equilibrium was zero.

This process of interpolating DD > 0°C, conversion to the expected number of ticks, and assigning a value for each CSD was repeated using downscaled output from the second version of the Coupled Global Climate Model (CGCM2) of the Canadian Centre for Climate Modelling and Analysis [32,33] as previously described [5]. We obtained output from the model that incorporated estimated forcing due to emissions of greenhouse and other gases calculated in emission scenario forcing '*A2*' for global economic change for the coming decades as defined by the Intergovernmental Panel on Climate Change [34]. Briefly, in scenario '*A2*', the future world is very much as it is at present in socio-economic terms: very heterogeneous with a continuously increasing population, regionally oriented economic development, and fragmented per capita economic growth and technological change [34]. DD > 0°C were calculated at each grid point by accumulating the daily positive mean temperature values throughout the whole year. In each case, the process was repeated for all 30 years of the three future time periods, and the climatic 'normal' of each period at each grid point was obtained by averaging 30 years of annual DD > 0°C. We therefore had interpolated surfaces of estimated *I. scapularis *abundance (assuming temperature was the only determinant of survival, as previously discussed [1,5,19]) at four time periods: the 'present day' (i.e. using recorded meteorological data for the period 1971 to 2000), and three future time slices centred around the 2020s, 2050s and 2080s.

#### Habitat

Habitat is a crucial determinant of *I. scapularis *survival [19,35]. All studies associate *I. scapularis *with woodland or woodland ecotone habitats [11,12,19,35], but clear associations with particular woodland types and other environmental indicators within a study (e.g. soil types and drainage) conflict amongst studies [e.g. [11,12,19]]. In general in the USA, coniferous forest is considered unsuitable habitat for *I. scapularis *and protective against establishment of the tick [11,12]. However in Nova Scotia, *I. scapularis *populations have established in coniferous woodland [unpublished L.R. Lindsay]. While there are likely to be common habitat factors that are fundamental to tick survival amongst all sites, it is possible that these have been overlooked in the studies to date. Habitat factors identified to date as risk factors for tick occurrence are possibly proxies for the real habitat determinants of tick survival. Because of this uncertainty, we simply assigned to each CSD the percentage forest cover in that CSD (excluding water bodies) to represent the proportion of potentially suitable habitat for *I. scapularis *and thus a potential determinant of *I. scapularis *population occurrence. Forest data based on AVHRR remote-sensed proxies for habitat were obtained from a Natural Resources Canada land cover map at a resolution of 1 km^2 ^[36] and percentage forest cover calculated for each CSD in ArcGIS.

#### Indices of tick immigration

The number of ticks immigrating into a particular CSD in Canada will be proportional to the number of *I. scapularis*-carrying birds that arrive in spring in that CSD, and the number of ticks that each bird carries. As described previously [20], a wide range of passerine species migrating into Canada each spring carry *I. scapularis*, and these birds fly along a wide range of routes, predominantly in a general South-North direction, but with much movement across country too [37]. Therefore, we constructed indices of tick immigration rates for each CSD as follows.

First, a map of US counties endemic for *I. scapularis *was created from the data in Dennis et al [26] using the georeferenced data file for US counties (the spatial resolution of the data in [26]) in ArcGIS, which was joined to a georeferenced data file for endemic Canadian CSDs. A 'buffer' with a radius of 425 km was then created for each CSD centred on the centroid of each CSD. A distance of 425 km was chosen because this is the mean distance that a passerine will fly in 5 days (the very maximum length of time that a nymphal *I. scapularis *would likely remain attached to a host: [10]), assuming a mean 85 km flown per day [21]. The number of US counties and Canadian CSDs endemic for *I. scapularis *within the 425 km radius from the centroid of each CSD was calculated and this became the index of immigration for nymphal *I. scapularis *for each CSD. While *I. scapularis *nymphs must feed for 4 days, the birds could stop in a CSD for longer periods due to inclement weather, or they could stop to establish breeding territory, so 425 km represented the maximum distance the ticks could be dispersed, but not the minimum distance they could be transported. By a similar method, an index of larval tick immigration was created using a buffer with a radius of 255 km, the maximum distance a larval tick could be transported were it to remain on a bird for three days.

The index of nymphal tick immigration was validated against data from passive surveillance for *I. scapularis *from 1990 to 2003 in which the same CSDs used in the current study formed the spatial unit [23]. Two methods of validation were used. First we used the whole database of locations of *I. scapularis *submitted by the public, via provincial organisations to the Public Health Agency of Canada, to compare how well the index of nymphal tick immigration predicted the number of ticks submitted by the public from each CSD in negative binomial regression models in STATA version 8.0 for Windows (STATACorp, College Station, TX, USA). Province of origin was included as a fixed effect because of differences amongst provinces in the effort expended in passive surveillance, and the natural logarithm of the human population in each CSD (as estimated in the 2001 census [24]) was an offset in the model, because the number of submissions inevitably depends on the human population density [23].

Second, we investigated the index of nymphal tick immigration as a predictor of the rate of submission of *I. scapularis *in the existing passive surveillance programme conducted in southern Quebec. Here surveillance has been most intense and long-lived, and risk factors for *I. scapularis *have already been identified [23]. These factors were mean spring NDVI (as a remotely-sensed proxy for forest cover) and latitude [23]. This earlier study raised the hypothesis that the observed geographic distribution of *I. scapularis *occurrence in southern Quebec was determined by the distance birds travel from endemic areas in the USA, a south-north temperature gradient (with the variable 'latitude' possibly acting as a proxy for one or both of these factors), and the suitability of habitat for bird-borne nymphal ticks to survive the moult to become questing adults. If so, then our indices of tick immigration should also partly explain the occurrence of *I. scapularis *in Quebec, and in particular be a confounder of 'latitude' as an explanatory variable. We therefore re-created the negative binomial regression model used in reference [23] in STATA (with the natural logarithm of the human population in each CSD as an offset), and tested whether the nymphal tick immigration index partly explained *I. scapularis *submissions, and to what extent confounding between the index of nymphal tick immigration and latitude occurred. The fit of the different models was compared using AIC [38].

### Selection of risk algorithms and risk map construction

While we have *a priori *information to suggest the dependence of *I. scapularis *distributions in Canada on combined effects of temperature, habitat and tick dispersion on migratory birds, we have no information on the relative magnitude of these effects. The small number of known *I. scapularis *populations in Canada provides little scope to explore these relationships in formal spatial logistic regression analyses, and obtain accurate estimates for the relative effects of different explanatory variables. Furthermore, here we also wished to identify locations that are not suitable for tick establishment (which is important in Lyme disease diagnosis [1]), which could arise if i) there were no immigrating ticks, irrespective of whether temperature or habitat were suitable, or ii) temperature was not suitable or there was no suitable habitat even though there may be many immigrating ticks. It is possible that multivariable logistic regression models could identify a location as suitable even if one explanatory variable alone predicts a very low probability for the occurrence of a tick population.

We therefore opted, as a first step in risk mapping, to use simple algorithms that combined the effects of these factors by multiplication into an index of risk for *I. scapularis *population establishment in Canada. A range of algorithms were chosen (Table 1), which predicted i) a risk index of zero for *I. scapularis *establishment in a CSD if the value for any one factor for that CSD was zero, or ii) a positive value obtained by multiplication of the different components of the algorithm. More complex non-linear relationships were not investigated due to the absence of *a priori *hypotheses. There was little correlation amongst the three components of these algorithms (number of ticks at model equilibrium, indices of tick immigration and percent forest cover). Pearson correlation coefficients were less than 0.21 in each pair wise comparison, with the exception that the correlation coefficients for the index of larval tick immigration and the number of ticks at model equilibrium, and the index of nymphal tick immigration were 0.58 and 0.57 respectively.

We then compared how well each algorithm predicted the occurrence of the 16 CSDs or partial CSDs in Canada in which, prior to our field validation, *I. scapularis *populations were known or suspected (i.e. under investigation) to have established since 2000 (one in Manitoba, three in Nova Scotia and the rest in Ontario), and 10014 presumed negative CSDs. Note that two populations (one in Nova Scotia and one in Ontario) straddle more than one CSD resulting in a greater number of *I. scapularis*-positive CSDs than there are known populations. All the *I. scapularis *populations have been identified, and their geographic scope determined, by a combination of data on passive surveillance for ticks and the occurrence of human cases, and intensive field studies using established criteria [1]. ROC analysis in STATA version 8.0 for Windows was used for these comparisons because it explicitly generates specificity and sensitivity values for a range of cut-off values [25] that can be used to select cut-off values for classifying CSDs for risk mapping purposes. We did, however, compare whether AUC values in ROC analysis and AIC values for logistic regression models gave similar results in terms of algorithm selection. Logistic regression models were created in STATA version 8.0 for Windows for each algorithm. In these models *I. scapularis *population occurrence was the outcome, and the components of the risk algorithms were the explanatory variables. We used ROC under the assumption that, for the most part known *I. scapularis *populations are spatially separated [1,20], and statistically independent of one another [39]. Predictions were made with only *I. scapularis*-endemic USA counties and Canadian CSDs known to contain tick populations prior to 2000 as source locations for immigrating ticks. Only data from CSDs east of 120°W (i.e. east of the Rocky Mountains) and south of 55°N were used in this analysis to limit the number of negative values that would be certainly predicted. Throughout this evaluation process, we used algorithm values in which the number of ticks was calculated from DD > 0°C values obtained from interpolations of 1971–2000 temperature normals.

The best performing algorithm (i.e. that having the highest area under the curve in ROC analysis [25]) was then used to develop risk maps. ROC was used for algorithm selection rather than specifically to estimate the accuracy of each algorithm. The Youden index (J = Sensitivity + Specificity - 1 [25]) was calculated for each of 1138 algorithm value points on the ROC curve. The algorithm value giving two thirds of the highest Youden Index was selected as the cut-off point above which CSDs were classified as likely to contain an *I. scapularis *population by the end of 2019 (i.e. belonging to what we termed the 'moderate risk' group of CSDs) assuming that temperature conditions do not change during the period 2000 to 2019. This cut-off value gave a sensitivity of 94% and specificity of 66% for detecting a CSD as likely to contain an *I. scapularis *population according to the ROC analysis. The 10% of the CSDs in the 'moderate risk' group with the highest algorithm values were re-classified as the 'high risk' group of CSDs for *I. scapularis *populations. The cut-off value for the 'high risk' group had a sensitivity of > 99% and specificity of < 1% for *I. scapularis *population occurrence according to ROC analysis. Three further groups were: i) low risk CSDs, i.e. those with an algorithm value > 1, but below the cut-off for inclusion in the 'moderate risk' group; ii) CSDs where there is no risk of *I. scapularis *population establishment (i.e. the algorithm value is zero) but those where there is a risk from bird-borne adventitious ticks (i.e. the value for the index of tick immigration > 1); and iii) a 'no risk' group of CSDs where both the algorithm value and the index of tick immigration were zero (i.e. CSDs outside the 425 km range of tick dispersion by migratory birds).

### Mapping risk under climate change scenarios

The selected algorithm could operate as a simple simulation model with 3 time steps. The starting point was a map of CSDs with a high index for *I. scapularis *population occurrence under current temperature conditions, which was combined with the map of US counties endemic for the tick [26]. This information provided a baseline map of the possible geographic range for *I. scapularis *prior to climate change.

The next time step is the 2020s, for which the index of nymphal immigration was recalculated but this time using a larger radius of 458 km because bird migration speed in North America is expected to increase with higher spring temperatures [21]. While the onset of migration of most migratory passerine species that nest in Canada may be controlled by endogenous circannual rhythms and photoperiod, the speed at which birds migrate north increases with increasing mean spring temperatures, possibly due to effects of temperature on the availability of the food the birds need for refuelling during their migration [21]. The change in speed (from 85 to 91.6 km per day) was estimated using the relationship observed between spring (April/May) temperatures in southern Ontario and Pennsylvania, and the speed with which migratory birds complete the journey from Louisiana to southern Ontario or Pennsylvania (for a 1°C increase in temperature, birds arrive one day earlier [21]). The temperature rise used to predict changes in migratory bird speed was the difference in mean spring (April/May) temperature for the Chatham, Ontario meteorological station during 1971–2000 and that temperature predicted for this station for the 2020s by downscaling and interpolation of CGCM2 output under IPCC emissions scenario *A2*. The maximum DD > 0°C (and thus the maximum number of ticks at model equilibrium) for each CSD also changed according to the interpolated, downscaled output from CGCM2 for the 2020s as described in the previous section on temperature. CSDs were then allocated to one of the five classes (high risk, moderate risk, low risk, risk from adventitious ticks and no risk) according to the new algorithm value obtained.

In turn, these populations, combined with pre-existing positive Canadian CSDs and US counties in the baseline, pre-climate change map, provided the 'source' of ticks for *I. scapularis *to establish in CSDs (that were previously free of the tick) by the 2050s. Again, the index of nymphal immigration was recalculated with the radius increased from 458 to 486 km, and the DD > 0°C (and thus the maximum number of ticks at model equilibrium) for each CSD changed according to the interpolated, downscaled output from CGCM2 for the 2050s. Each CSD was then re-classified in terms of risk of *I. scapularis *population establishment, and the process was repeated again for the 2080s with increasing radius of bird movement (from 486 to 532 km), DD > 0°C and modeled tick numbers using CGCM2 output for the 2080s.

At present, the true geographic extent of reproducing *I. scapularis *populations in Canada is uncertain, at least in part because the number of newly established populations is increasing and active surveillance for these populations to date has limited scope and is incomplete. For this reason, we ran the 'model' using two different scenarios: i) the 'fast' scenario in which a CSD was considered to contain an *I. scapularis *population (and would thus act as 'source' for the ticks) if the CSD were in the 'moderate risk' or 'high risk' groups, and ii) the 'slow' scenario in which a CSD was considered to contain an *I. scapularis *population only if the CSD were in the 'high risk' group.

### Field validation of risk maps under current climate conditions

Validation in the field was carried out in southern Quebec. We aimed to visit 70 sites (30 sites in 'moderate risk' CSDs and 40 sites in 'high risk' CSDs) giving 90% power to identify a difference in the possible prevalence of *I. scapularis*-infested sites predicted by the risk algorithm of 66% and 99% in moderate risk and high risk regions respectively with α = 0.05. However, for a number of reasons including flooding, clear cutting of trees, lack of permission and a total absence of trappable rodents, data were collected from 45 sites which gave a power of 75% to detect such a difference in prevalence. Teams of two researchers visited each site once from 5^th ^June to 5^th ^October 2007. Sites were selected to fulfill the following criteria: one site per CSD in two regions (Montérégie and Estrie) determined by the practicality of traveling distances, and deciduous woodlands (determined from provincial government forest cover maps [40]) of minimal dimensions 500 m by 150 m. Sites were visited in a haphazard fashion depending on contact with, and permission to study from, the landowners.

At each visit, rodents were trapped and host-seeking ticks were collected according to the following protocol. At each site 150 Sherman traps were placed in three parallel transects of 50 traps. The transects were placed 25–50 m apart, and within a transect, the traps were set 10 m apart. Traps were furnished with bedding and a mix of nuts, seeds and peanut butter as bait. Traps were set for two nights or one night if 15 or more rodents were captured in the first night (with α = 0.05, a sample of 15 rodents gives 80% power to identify an infested rodent if the true prevalence of infestation is 5%). All captured rodents were examined for the presence of ticks after light anaesthesia as described elsewhere [41]. The herbage of each site was flagged to collect host-seeking ticks for 3 person-hours in the afternoon. All ticks collected from rodents and herbage were identified to species by standard keys [42,43].

An 'index of certainty' that a reproducing population of *I. scapularis *ticks was present was determined for each site. Increasing values for this index gave increased confidence that ticks found at a site were from a reproducing population of ticks, rather than just bird-transported 'adventitious' ticks [20,23]. The index was calculated for each site as follows: presence of larvae = 1 point; presence of 3 or more larvae = 1 additional point; presence of 10 or more larvae = 1 additional point; presence of nymphs = 1 point; presence of 3 or more nymphs = 1 additional point; presence of 10 or more nymphs = 1 additional point; presence of adult ticks = 1 point; presence of 3 or more adults = 1 additional point; presence of 10 or more adults = 1 additional point. Thus the index had a minimum value of 0 (no *I. scapularis *found at the site) and a maximum of 9 (when 10 or more ticks of each of the three instars were found at the site). All ticks found on flagging and on rodents were included in this calculation.

The index of certainty was then the outcome in ordered logistic regression models which make no a priori assumptions as to the linearity or otherwise of the index, simply assuming that 1 is 'greater' than 0, 2 'greater' than 1 etc. [44]. Site classification (i.e. high risk or moderate risk), the value of the algorithm for the CSD in which the site occurred, and, independently, the tick immigration index and number of ticks at model equilibrium on which the algorithm for that CSD was based, were explored as predictors of the occurrence of *I. scapularis*. The fit of the different models in predicting the occurrence of likely *I. scapularis *populations was investigated using AIC. Potential confounding explanatory variables were explored including the month the site was visited (because different *I. scapularis *instars are active in different seasons [10]), the ID for the field team and the number of rodents captured at the site.

## Competing interests

The authors declare that they have no competing interests.

## Authors' contributions

NHO principal researcher and author.

LSO, SB, PM and RAT were responsible for GIS advice, operations and map production.

LRL, FM, LT and NHO were responsible for field validation.

LRL and LT were responsible for passive tick surveillance data.

NHO, MB-P, DFC and CJO were responsible for modelling *I. scapularis *populations.

LRL and IKB provided most of the data for the *I. scapularis *population model.

AM provided downscaled projected temperatures for Canada.

CMF and AH were responsible for data and information on bird migration.

All authors contributed to the MS.
